# Minoxidil Promotes Hair Growth through Stimulation of Growth Factor Release from Adipose-Derived Stem Cells

**DOI:** 10.3390/ijms19030691

**Published:** 2018-02-28

**Authors:** Nahyun Choi, Soyoung Shin, Sun U. Song, Jong-Hyuk Sung

**Affiliations:** 1 College of Pharmacy, Yonsei University, Incheon 21983, Korea; nh147837@gmail.com; 2 STEMORE Co., Ltd., Incheon 21983, Korea; 3 College of Pharmacy, Wonkwang University, Iksan 54538, Jeonbuk, Korea; shins@wku.ac.kr; 4 Translational Research Center and Inha Research Institute for Medical Sciences, Inha University School of Medicine, Incheon 21983, Korea

**Keywords:** minoxidil, adipose-derived stem cells, hair growth, CXCL1, PD-ECGF, PDGF-C

## Abstract

Minoxidil directly promotes hair growth via the stimulation of dermal papilla (DP) and epithelial cells. Alternatively, there is little evidence for indirect promotion of hair growth via stimulation of adipose-derived stem cells (ASCs). We investigated whether minoxidil stimulates ASCs and if increased growth factor secretion by ASCs facilitates minoxidil-induced hair growth. Telogen-to-anagen induction was examined in mice. Cultured DP cells and vibrissae hair follicle organ cultures were used to further examine the underlying mechanisms. Subcutaneous injection of minoxidil-treated ASCs accelerated telogen-to-anagen transition in mice, and increased hair weight at day 14 post-injection. Minoxidil did not alter ASC proliferation, but increased migration and tube formation. Minoxidil also increased the secretion of growth factors from ASCs, including chemokine (C-X-C motif) ligand 1 (CXCL1), platelet-derived endothelial cell growth factor (PD-ECGF), and platelet-derived growth factor-C (PDGF-C). Minoxidil increased extracellular signal–regulated kinases 1/2 (ERK1/2) phosphorylation, and concomitant upregulation of *PD-ECGF* and *PDGF-C* mRNA levels were attenuated by an ERK inhibitor. Subcutaneous injection of CXCL1, PD-ECGF, or PDGF-C enhanced anagen induction in mice, and both CXCL1 and PDGF-C increased hair length in ex vivo organ culture. Treatment with CXCL1, PD-ECGF, or PDGF-C also increased the proliferation index in DP cells. Finally, topical application of CXCL1, PD-ECGF, or PDGF-C with 2% minoxidil enhanced anagen induction when compared to minoxidil alone. Minoxidil stimulates ASC motility and increases paracrine growth factor signaling. Minoxidil-stimulated secretion of growth factors by ASCs may enhance hair growth by promoting DP proliferation. Therefore, minoxidil can be used as an ASC preconditioning agent for hair regeneration.

## 1. Introduction

Adipose-derived stem cells (ASCs) have stimulatory effects on dermal papilla (DP) cells to promote hair-growth [1,2,3,4,5,6]. For example, ASCs secrete multiple growth factors, such as vascular endothelial growth factor (VEGF) and basic fibroblast growth factors (bFGF), which can increase the proliferation of DP cells [1]. Festa et al. showed that adipocyte lineage cells drive hair cycling by contributing to the skin stem cell niche, and suggested that platelet-derived growth factor-A (PDGF-A) expression by immature adipocytes regulates follicular stem cell activity [2]. Grafting of ASC-enriched adipose tissue (i.e., by injection of the stromal vascular fraction of lipoaspirate) has shown promise as an alternative approach to treating baldness in men and women [7]. We have identified stimulators that enhance the hair-regenerative potential of ASCs in vivo. For instance, vitamin C and low-dose ultraviolet B (UVB) increased secretion of hair growth-promoting factors by ASCs and induced anagen in animal models [3,4]. Of the potential stimulators tested, we found that platelet-derived growth factor-D (PDGF-D) exhibited the strongest effects on ASCs and increased secretion of growth factors via mitogen-activated protein kinase (MAPK) pathways [5]. In addition, *CAP-18* for cathelicidin antimicrobial peptide (LL-37) increased the secretion of growth factors and the hair-regenerative efficacy of ASCs via early growth response 1 (ERG1) protein and the MAPK pathway [8].

Minoxidil was first developed to treat male- and female-pattern alopecia. In addition to vasodilation, there is strong evidence that minoxidil directly promotes hair growth via the stimulation of DP and epithelial cells [9,10,11,12,13,14]. Minoxidil stimulated mouse vibrissae follicles in organ culture and induced proliferation of hair epithelial cells near the follicle base [9]. Further, minoxidil and its derivatives showed cytoprotective activity in vivo and increased prostaglandin E2 (PGE2) production by human DP fibroblasts [11]. In cultured DP cells, minoxidil-induced hair growth was mediated by adenosine receptors [12]. Minoxidil also promoted the survival of human DP cells by activating both the ERK and protein kinase B (Akt) pathways, and prevented apoptotic cell death by increasing the ratio of Bcl-2/Bax [15]. Moreover, minoxidil activated the β-catenin pathway in human DP cells, suggesting a possible mechanism for its anagen prolongation effect [13]. Minoxidil suppressed androgen receptor (AR)-mediated functions by decreasing AR transcriptional activity in reporter assays and reducing expression of AR targets at the protein level [16]. Otomo summarized the primary mechanisms of minoxidil action as (a) induction of growth factors in DP cells, such as VEGF, hepatocyte growth factor (HGF), and insulin-like growth factor-1 (IGF-1); (b) inhibition of TGF-β-induced apoptosis of hair matrix cells; and, (c) increase of blood flow by dilating hair follicle arteries [14]. However, there is little evidence that minoxidil can indirectly promote hair growth via ASCs, even though ASCs contribute to the stem cell niche for hair follicles and exert stimulatory effects on hair cycle progression. Therefore, in the present study, we examined possible indirect hair growth-promoting effects of minoxidil via ASCs. Specifically, we investigated whether minoxidil stimulates growth factor secretion by ASCs to enhance follicular cell activity and hair growth. 

## 2. Results

### 2.1. Minoxidil-Pretreated Adipose-Derived Stem Cells (ASCs) Promote Hair Growth In Vivo 

**Figure 1:**
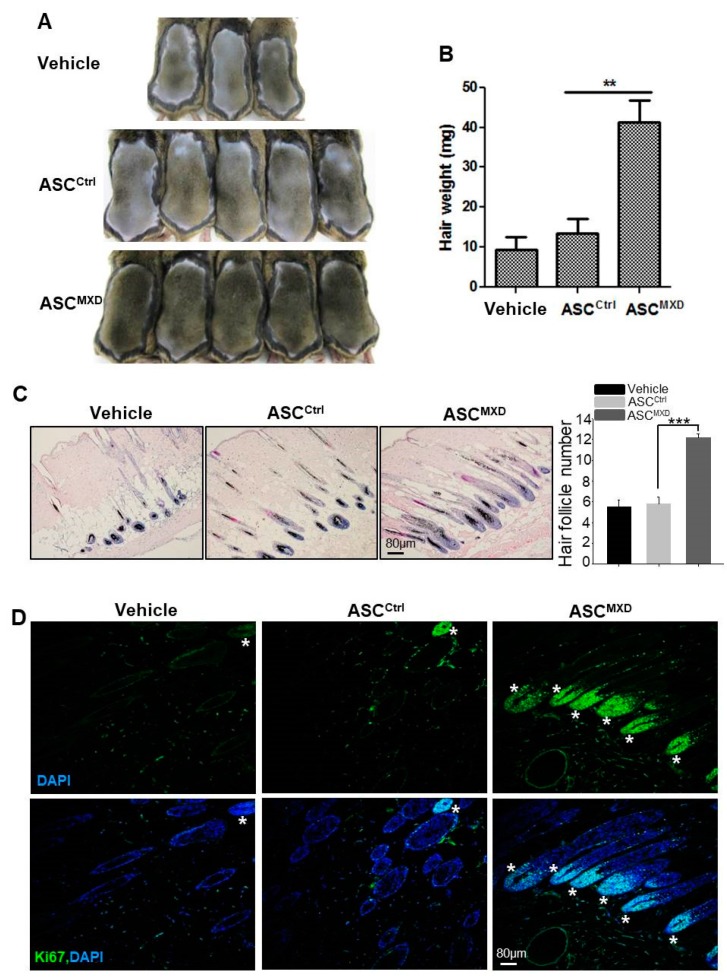
Adipose-derived stem cells (ASCs) pretreated with minoxidil promote hair growth in vivo. Minoxidil-treated ASCs or untreated ASCs were injected into the dorsal skin of shaved mice. Photograph was taken (**A**), and hair weight measured (**B**) 14 days later; (**C**) Skin section was analyzed by HE staining and the number of mature hair follicle was measured; (**D**) The hair follicle with Ki67^+^ cells in DP was shown by immunostaining. Asterisks indicate hair follicles with Ki67^+^ DP cells. ** *p* < 0.01, *** *p* < 0.001. *n* = 7 or 8 mice per group. All error bars indicate SEM.

ASCs are known to stimulate hair growth [1,2,3,4,5,6]. We found that injection of naïve (untreated) human ASCs increased telogen-to-anagen induction in mice only slightly following subcutaneous injection, while ASCs pretreated with minoxidil induced robust hair growth (Figure 1A,B). To examine the effect of ASCs pretreated with minoxidil on hair follicle, we performed hematoxylin and eosin (HE) staining and immunofluorescence staining for Ki67, which is a proliferating cell marker in DP. The skin section of ASC^MXD^-treated mice showed higher number of mature hair follicle compared to vehicle- or ASC^Ctrl^-treated mice (Figure 1C). In addition, most hair follicles of ASC^MXD^-treated mice showed DP with Ki67^+^ cells contrary to vehicle- or ASC^Ctrl^-treated mice (Figure 1D). This result suggests that minoxidil can promote telogen to anagen induction, thereby promoting hair growth. 

### 2.2. Minoxidil Can Induce Migration of ASCs 

**Figure 2:**
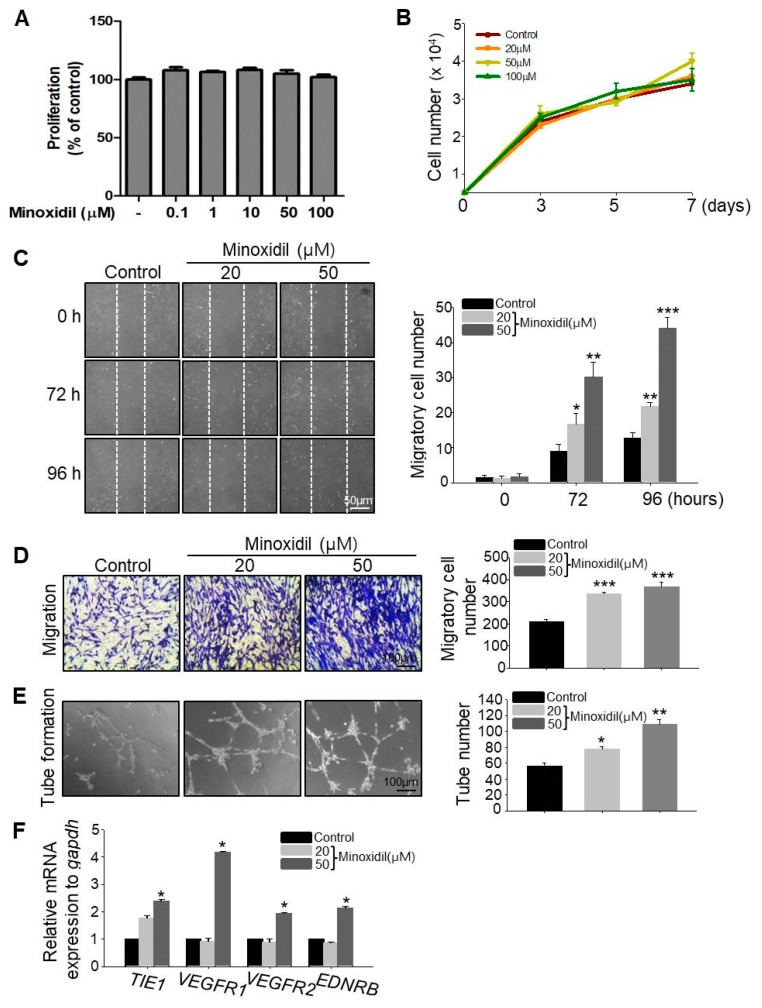
Minoxidil promotes ASC migration and tube formation but not proliferation. (**A**,**B**) No effect of minoxidil on proliferation of ASCs. (**C**,**D**) Minoxidil enhances ASC migration as evidenced by both scratch migration assay (**C**) and transwell migration assay (**D**); (**E**) Tube formation assay showing enhanced formation by minoxidil-treated ASCs. (**F**) Relative mRNA expression levels of endothelial cell markers including *TIE1, VEGFR1, VEGFR2*, and *EDNRB* genes were increased in tubes from minoxidil-treated ASCs. * *p* < 0.05, ** *p* < 0.01, *** *p* < 0.001. Three independent experiments were conducted per data point. All error bars indicate SEM.

To examine whether minoxidil affects ASC proliferation, we determined the live cell number over two days and seven days of minoxidil treatment. Minoxidil had no effect on ASC proliferation under either condition, even at the highest dose (Figure 2A,B). To explore whether minoxidil affects ASC migration, we conducted scratch and transwell migration assays. Minoxidil at 20 and 50 µM dose-dependently increased ASC migration into both the scratch wound assay (Figure 2C) and transwell migration assay (Figure 2D). To determine whether the effect of minoxidil on specific to the ASCs, we examined the effect of minoxidil on growth and migration of dermal fibroblast cells. Minoxidil did not induce cell growth either migration (Figure S1). Initially, topically applied minoxidil was believed to stimulate hair growth by indirect actions, such as vasodilatation and increased blood flow to the DP [10,14]. Therefore, we examined whether minoxidil affects blood vessel formation by ASCs using an in vitro tube formation assay. Indeed, minoxidil dose-dependently increased the number of nascent tubes after 12–16 h (Figure 2E) and the expression level of endothelial cell markers including tyrosine kinase with immunoglobulin-like and EGF-like domains 1 (*TIE1*), vascular endothelial growth factor receptor 1 (*VEGFR1*), *VEGFR2* and endothelin receptor type B (*EDNRB*) (Figure 2F). Collectively, these results suggest that minoxidil may promote hair growth by enhancing ASC migration and ASC-dependent angiogenesis.

### 2.3. CXCL1, ECGF, and PDGF-C Induce Hair Growth

**Figure 3:**
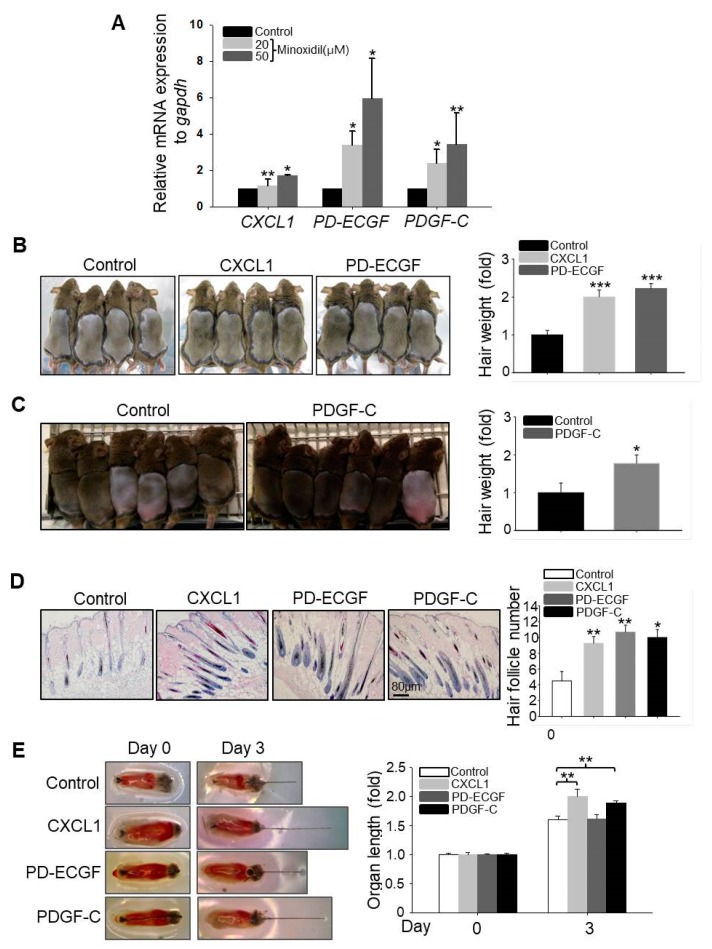
Minoxidil upregulates expression of hair-growth promoting factors CXCL1, PD-ECGF, and PDGF-C in ASCs. (**A**) Relative mRNA expression levels of growth factors including *CXCL1*, *PD-ECGF*, and *PDGF-C* were measured in minoxidil-treated ASCs; (**B**–**D**) Human recombinant CXCL1, PD-ECGF, and PDGF-C proteins each facilitated hair growth and increased the number of mature hair follicle after injection into the dorsal skin of shaved mice as revealed by hair weight at 2 weeks post-injection. *n* = 6–8 mice per group. * *p* < 0.05, ** *p* < 0.01, *** *p* < 0.001. (**E**) CXCL1, PD-ECGF, and PDGF-C proteins each facilitate mouse vibrissal hair follicle growth ex vivo. ** *p* < 0.01. *n* = 10 samples per treatment group. All error bars indicate SEM.

It has been reported that growth factors secreted by ASCs, such as VEGF, fibroblast growth factor-1 (FGF1), bFGF, and PDGF-A, regulate hair follicular stem cell activity and induce the anagen phase of the hair cycle in vivo [14]. We speculated that minoxidil may promote hair growth indirectly by enhancing growth factor release from ASCs. To explore this possibility, we compared expression patterns between untreated naïve and minoxidil-treated ASCs by qPCR array. Minoxidil upregulated the expression of PD-ECGF over six-fold when compared to untreated ASCs (Figure S2), and this result was confirmed (Figure 3A). Moreover, minoxidil also upregulated the expression levels of *CXCL1* and *PDGF-C* (Figure 3A).

To investigate whether CXCL1, PD-ECGF, and PDGF-C can induce the anagen phase of the hair cycle in vivo, we injected recombinant human CXCL1, PD-ECGF, or PDGF-C protein into the subcutaneous dermis of shaved mice. All three factors significantly induced the anagen phase of the hair cycle in vivo (Figure 3B,C) and increased the number of mature hair follicle (Figure 3D), suggesting that minoxidil may induce anagen by triggering CXCL1, PD-ECGF, or PDGF-C release from ASCs. Moreover, treatment with CXCL1 or PDGF-C, but not PD-ECGF, also increased the length of isolated mouse vibrissal hair follicles in organ culture (Figure 3E). These results strongly suggest that minoxidil promotes hair growth through growth factor release from ASCs. 

### 2.4. Minoxidil Regulates Expression of PD-ECGF and PDGF-C in ASCs through the ERK Pathway

**Figure 4:**
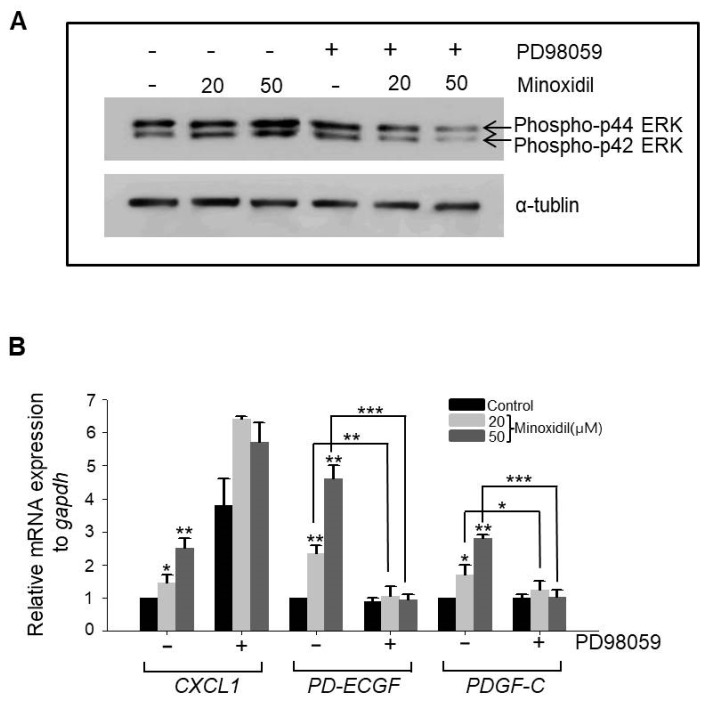
Minoxidil upregulates expression of *PD-ECGF* and *PDGF-C* through the ERK pathway in ASCs. (**A**) The MEK inhibitor PD98059 reversed minoxidil-induced ERK phosphorylation; (**B**) PD98059 also suppressed minoxidil-induced upregulation of *PD-ECGF* and *PDGF-C* expression by ASCs. Three independent experiments were carried out per data point. * *p* < 0.05, ** *p* < 0.01, *** *p* < 0.001. All the error bars indicate SEM.

It has been reported that the MAPK pathway regulates expression of growth factors in ASCs, including vascular endothelial growth factor A (VEGFA) and FGF1 [5,8]. Therefore, we examined whether minoxidil regulates the expression of CXCL1, PD-ECGF, or PDGF-C through the MAPK pathway. Indeed, minoxidil dose-dependently upregulated phospho-ERK expression, a response suppressed by the specific mitogen-activated protein kinase kinase (MEK) inhibitor PD98059 (Figure 4A). Further, PD98059 reversed minoxidil-induced upregulation of PD-ECGF and PDGF-C in ASCs (Figure 4B). Alternatively, minoxidil-induced upregulation of CXCL1 was not affected by PD98059 (Figure 4B), suggesting that minoxidil upregulates growth factor expression in ASCs through multiple pathways, including the MAPK pathway.

### 2.5. ASC Growth Factors CXCL1, PD-ECGF, and PDGF-C Induce DP Cell Proliferation 

**Figure 5:**
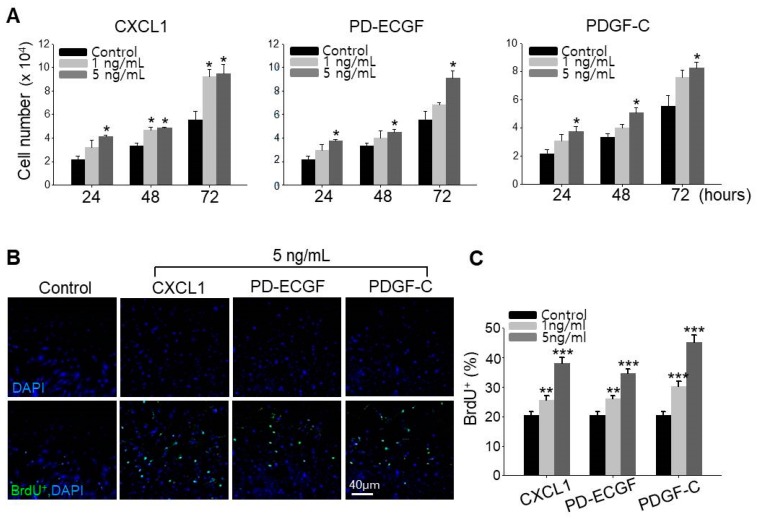
CXCL1, PD-ECGF, and PDGF-C induce proliferation of DP cells. (**A**) Cell growth was measured after treatment of CXCL1, PD-ECGF, or PDGF-C protein in DP cells for 3 days. Three independent experiments were conducted. * *p* < 0.05. (**B**,**C**) Proliferation index (% of BrdU^+^ cells; green) after treatment of CXCL1, PD-ECGF, or PDGF-C protein in DP cells. Three independent experiments were carried out per data point. ** *p* < 0.01, *** *p* < 0.001. All error bars indicate SEM.

It has been reported that minoxidil stimulates the growth of human hairs by prolonging anagen through proliferative and anti-apoptotic effects on DP cells [13,15]. We therefore directly examined whether CXCL1, PD-ECGF, or PDGF-C increase DP cell proliferation, and indeed, all three factors when applied separately dose-dependently increased cultured DP cell proliferation (Figure 5A). To confirm this observation is specific to DP cells, we examined whether CXCL1, PD-ECGF, or PDGF-C increase the proliferation of human dermal fibroblast cells. All three proteins did not induce the proliferation of fibroblast contrary to increase in DP cells (Figure S3). Further, all three factors enhanced the number of DP cells in S-phase as evidenced by 5-bromo-2′-deoxyuridine (BrdU) labeling. Moreover, treatment of CXCL1, PD-ECGF, or PDGF-C in DP cells also dose-dependently increased the percentage of BrdU^+^ cells, which is proliferation index (Figure 5B,C). These results further suggest that minoxidil may enhance DP proliferation indirectly by inducing release of growth factors, such as CXCL1, PD-ECGF, and PDGF-C from ASC.

### 2.6. Application of CXCL1, PD-ECGF, or PDGF-C Acts Synergistically with Minoxidil to Induce Hair Growth

**Figure 6:**
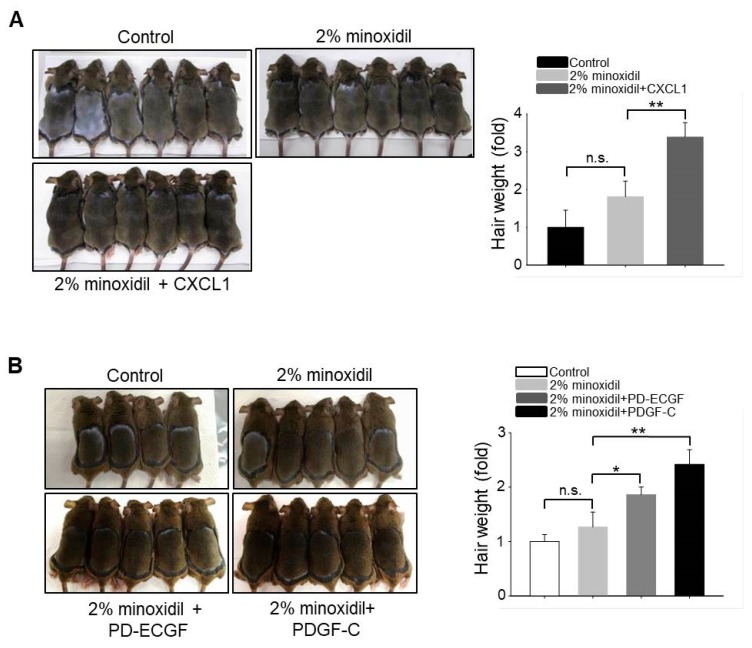
Application of CXCL1, PD-ECGF, or PDGF-C enhances the hair growth-promoting effect of minoxidil (2%) when co-applied on the dorsal skin of shaved mice. (**A**) Photograph was taken and hair weight was measured 14 days later for CXCL1 application. ** *p* < 0.01. *n* = 6 mice per group; (**B**) Photograph was taken and hair weight was measured 14 days later for PD-ECGF or PDGF-C application. * *p* < 0.05, ** *p* < 0.01. n.s. indicates not significant. *n* = 5 or 6 mice per group. All of the error bars indicate SEM.

Our results suggest that upregulation of CXCL1, PD-ECGF, or PDGF-C by minoxidil stimulates DP cell proliferation, resulting in hair growth. Minoxidil has been widely used to treat androgenetic alopecia. Therefore, to investigate whether application of CXCL1, PD-ECGF, or PDGF-C acts synergistically with 2% minoxidil to induce the anagen phase of the hair cycle, we compared hair growth on the dorsal skin among shaved mice that were treated with 2% minoxidil alone or minoxidil plus either CXCL1, PD-ECGF, or PDGF-C for 14 days. Co-administration of each protein increased hair weight when compared to 2% minoxidil alone (Figure 6A,B), suggesting that the addition of these proteins may enhance the efficacy of minoxidil.

## 3. Discussion

**Figure 7:**
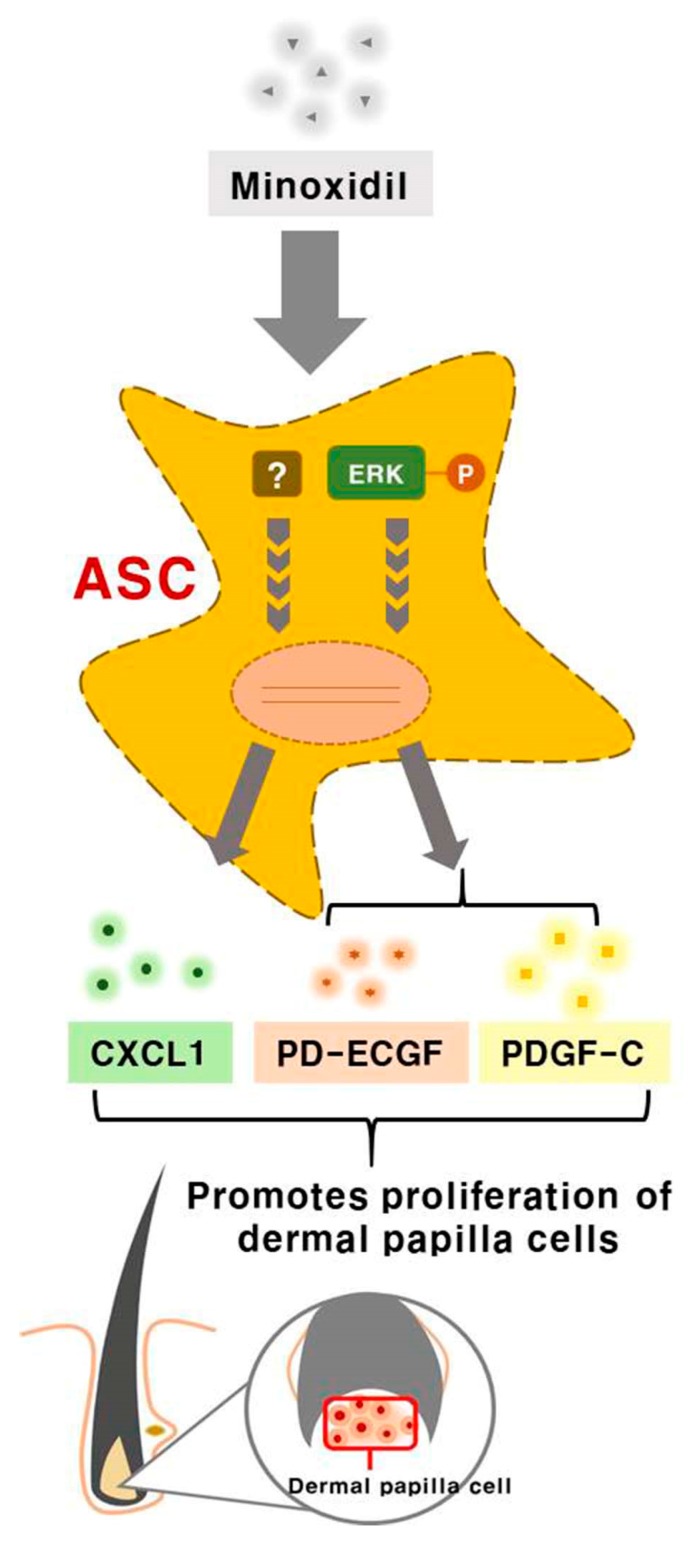
Minoxidil promotes the proliferation of DP cells and hair growth through stimulation of growth factor release from adipose-derived stem cells. Minoxidil stimulates the release of growth factors including PD-ECGF, PDGF-C, and CXCL1 from ASCs via ERK and the other pathway, respectively, thereby promoting of DP cells proliferation and hair growth.

Minoxidil promotes hair growth directly by stimulating DP and epithelial cells, but previous studies provided little or no evidence for indirect hair growth-promoting effects through the stimulation of ASCs. Therefore, we investigated whether minoxidil stimulates ASCs and enhances hair growth through growth factor release. We first demonstrated that subcutaneous injection of minoxidil-treated ASCs accelerated telogen-to-anagen transition in C3H/HeJ mice and increased hair weight after two weeks. Although minoxidil did not alter the proliferation of ASCs, it did increase migration, tube formation, and secretion of growth factors, notably CXCL1, PD-ECGF, or PDGF-C (the later two through ERK activation). Further, each of these growth factors enhanced DP cell proliferation in vitro and anagen induction in mice, while CXCL1 and PDGF-C also increased the length of isolated mouse vibrissal hair follicles in organ culture. Moreover, these hair growth-promoting effects were synergistic with 2% minoxidil. Collectively, these results indicate that minoxidil stimulates hair growth in part through stimulation of CXCL1, PD-ECGF, or PDGF-C release from ASCs (Figure 7).

Chemokine (C-X-C motif) ligand 1 is a secreted growth factor that signals through the G-protein coupled CXC receptor 2, while CXCL1 is known to act as a potent chemoattractant for neutrophils during inflammation [17,18,19]. However, it has not been linked to hair regeneration. PD-ECGF promotes angiogenesis in vivo and stimulates the in vitro growth of multiple endothelial cell types [20,21,22]. It has a highly restricted target cell specificity, acting only on endothelial cells. Of interest, *PD-ECGF* was one of the top three genes that were upregulated in ASCs by minoxidil according to qPCR arrays. Moreover, our work in vitro and in vivo revealed that minoxidil-induced PD-ECGF release from ASCs, resulting in DP proliferation and hair growth. On the contrary, it has been well-known that PDGF signaling in the dermis and in dermal condensates is dispensable for hair follicle induction and formation [23]. For example, PDGF-A secreted from ASCs regulates follicular stem cell activity [2]. PDGF-A and -B are involved in the induction and maintenance of the anagen phase in the mouse hair cycle [24]. Our work revealed that PDGF-C also functions in hair growth by upregulating DP proliferation. Although these results might be expected because many growth factors are already known to promote hair cycling, upregulation by minoxidil is meaningful for further usability of this agent to treat androgenetic alopecia. Indeed, the co-application of PDGF-C, CXCL1, or PD-ECGF with 2% minoxidil synergistically increased hair growth (Figure 6). 

It has been reported that PDGF-D treatment induces growth factor secretion from ASCs via phospho-activation of ERK [5,8]. Meldrum’s group also reported that preconditioning (i.e., by hypoxia or transforming growth factor) induced the secretion of numerous growth factors from mesenchymal stem cells through the MAPK pathways [25,26,27,28]. Similarly, our work revealed that minoxidil induced PD-ECGF and PDGF-C release from ASCs via ERK phosphorylation (Figure 4). Therefore, it is reasonable to assume that growth factor secretion by ASCs is primarily regulated by the MAPK pathway. However, it appears that CXCL1 is induced via an ERK-independent pathway, suggesting that minoxidil may activate multiple signaling pathways in ASCs. Further investigation is required to identify the pathway mediating minoxidil-induced CXCL1 release.

Minoxidil reportedly increased the proliferation of DP cells in vitro. For example, minoxidil promoted the survival of human DP cells by activating both ERK and Akt pathways, and prevented cell death by increasing the ratio of Bcl-2/Bax [15]. In addition, minoxidil plus all-trans retinoic acid (ATRA) additively promoted hair growth in human hair follicle culture [29]. A combination of minoxidil with ATRA elevated phosphorylated ERK, phosphorylated Akt in DP cells and keratinocytes [29]. However, minoxidil did not increase the proliferation in ASCs, which indicates that proliferative effect of minoxidil is cell type dependent.

ACSs are considered an important component of other stem cell niches. Indeed, it has been shown that adipocyte lineage cells are part of the skin stem cell niche that drives hair cycling, and it was suggested that PDGF-A expression by immature adipocytes is a key regulator of follicular stem cell activity [2]. Although we did not investigate the effects of minoxidil or ASC-released growth factors on DP stem cells, ASC-released growth factors (specifically CXCL1, PD-ECGF and PDGF-C) induced DP cell proliferation, consistent with a pivotal role in the regulation of DP stem cells. To determine whether the effect of minoxidil on specific to the ASCs, minoxidil didn’t induce cell growth either migration of dermal fibroblast cells (Figure S1).

We previously reported that preconditioned ASCs by growth factor, such as PDGF-D, enhance hair generative potential in tellogen to anagen induction model [5]. The purpose of this model is to induce anagen phase more quickly after we injected ASCs by paracrine effect. Anagen hair induction was not limited to the injection site. Instead, darkening of the skin or hair appeared across all areas of the back. We showed increased hair weight in ASC^MXD^-treated mice, which indicates the increase of hair length as well as hair number. The measurement of hair weight as an evaluation for hair growth is not enough to conclude increased telogen-to-anagen induction. Therefore, we examined hair follicle and proliferating DP cells in sectioned skin. The skin section of ASC^MXD^-treated mice showed higher number of mature hair follicle compared to vehicle- or ASC^Ctrl^-treated mice (Figure 1C). In addition, most hair follicles of ASC^MXD^-treated mice showed DP with Ki67^+^ cells contrary to vehicle- or ASC^Ctrl^-treated mice (Figure 1D). This result suggests that minoxidil can promote telogen to anagen induction, thereby promoting hair growth. Although the measurement of hair weight is not enough to examine the growth of hair follicle and DP proliferation, this method is easy to monitor hair growth quickly. 

In summary, subcutaneous injection of minoxidil-treated ASCs accelerated the telogen-to-anagen transition in mice, and direct minoxidil treatment increased migration, tube formation, and growth factor secretion by ASCs. The most strongly upregulated growth factors, CXCL1, PD-ECGF and PDGF-C, individually enhanced anagen induction in mice, while CXCL1 and PDGF-C also increased the length of isolated mouse vibrissal hair follicles in organ culture. Therefore, minoxidil can be used as a novel ASC preconditioning agent for hair regeneration.

## 4. Materials and Methods 

### 4.1. Cell Culture

Human adipocyte-derived stem cells (ASCs) were isolated via liposuction of subcutaneous fat as described previously [30,31]. Briefly, fat was washed with phosphate-buffered saline (PBS), added 0.075% collagenase and incubated for 45 min at 37 °C with gently shaking. The pellet after centrifugation was filtered with 100 µm nylon mesh, gathered again after centrifugation. Then, cells were cultured with essential medium, including α-minimum essential media (MEM) (Hyclone, Logan, UT, USA), 10% fetal bovine serum (Gibco, Carlsbad, CA, USA) and 1% anti-antibiotics (Gibco) until passage 3. Then, medium was changed to minimum essential medium including α-MEM, 10% fetal bovine serum, and 1% penicillin/streptomycin (Gibco) at four passages. ASCs were used at passages 5–7 for all of the experiments. Characterization of ASCs was performed using flow cytometry. ASCs were positive for CD44, CD73, CD90, CD105, human leukocyte antigen-I (HLA-I), and podocalyxin (PODXL), but were negative for hematopoietic markers such as CD34 and CD45 [32,33]. Multipotent differentiation potential was examined as described previously [34,35], and ASCs could be differentiated into adipocytes, osteocytes, and chondrocytes. We purchased human DP cells from PromoCell (#C-12071, PromoCell, Logan, Heidelberg, Germany). Human DP cells were cultured in Follicle DP Cell Medium (C-26505; PromoCell) with SupplementMix (C-39625; PromoCell) and 0.1% anti-antibiotics (Gibco). DP cells were used at passages 3, 4. We also purchased human dermal fibroblast cells from PromoCell (#C-12302). Dermal fibroblasts were cultured in dulbecco modified eagle medium (DMEM)/high glucose (Hyclone) with 10% fetal bovine serum and 1% penicillin/streptomycin, and were used at passages 8–10. All of the cells were maintained at 37 °C in a humidified 5% CO_2_ incubator (Theremo Fisher scientific, Waltham, MA, USA). 

### 4.2. Cell Growth Assay

For measuring the effects of minoxidil on ASC proliferation, cells were seeded in 6-well plates at 1 × 10^4^ cells/well, treated with minoxidil (0.1–100 µM), and incubated for two days in an InCu-saFe CO_2_ incubator (Panasonic, Kadoma, Osaka, Japan). Live cell number was determined using the IncuCyte zoom2014A live cell analysis system (Panasonic, Kadoma, Osaka, Japan) [5]. Alternatively, ASCs were seeded in 12-well plates at 5000 cells/well, treated with minoxidil (20, 50, or 100 µM), and incubated for seven days. Cells were then trypsinized, stained with Trypan Blue (Sigma-Aldrich, St. Louis, MO, USA), and counted each day using a hemocytometer hemocytometer. To assess the effects of growth factors secreted by ASCs on DP cell proliferation, 1 × 10^4^ DP cells were seeded in 12-well plates and treated with the indicated synthesized peptides for up to three days. Cells were trypsinized and stained with Trypan Blue. Viable cell number was counted each day using a hemocytometer. 

### 4.3. Scratch Migration Assay 

Cells were seeded into 6-well plates and cultured to confluence. A sterile 1 mL pipette tip was used to scratch the cell monolayer. Cultures were then washed with PBS to removed deplated cells and cultured with minoxidil in serum-free medium for four days. Migration of cells into the scratched area (wound closure) was visualized using a ZEISS Observer D1 microscope (Carl ZEISS, Oberkochen, Germany). Multiple images were acquired per well and average cell counts within the wound monitored over 4 days. 

### 4.4. Cell Migration Assay Using Transwell

ASCs (1 × 10^4^/well) were suspended in serum-free medium and seeded on the upper side of transwell membrane plates (BD falcon, BD Biosciences, San Jose, CA, USA). After 2 h, minoxidil in serum-free medium were introduced into the lower chambers. Cultures were incubated for two days to allow transwell migration. Inserts were then removed and the upper surface was cleaned of non-migrating cells using cotton swabs and washed with PBS. The inserts were stained with 0.1% formalin/10% crystal-violet solution (Sigma-Aldrich) and cell number analyzed under a ZEISS Observer D1 microscope. Multiple images (15–20) per insert were acquired, and average cell counts were calculated.

### 4.5. Tube Formation Assay Using Matrigel

Twelve-well plates were coated with matrigel (BD matrigel matrix, BD Biosciences) and were dried for 2 h at 37 °C. ASCs in endothelial cell basal medium-2 (EBM-2, LONZA, Walkersville, MD, USA) plus minoxidil were plated on matrigel-coated wells and incubated for 12–16 h at 37 °C. The number of tubes was analyzed under a ZEISS Observer D1 microscope [3], and the expression level of endothelial cell markers was analyzed by qPCR.

### 4.6. RNA Extraction and Quantitative RT-PCR

Total RNA was extracted from ASCs using Trizol reagent (Invitrogen, Carlsbad, NY, USA) and subjected to cDNA synthesis using oligodT and the HelixCript™ Thermo Reverse Transcription System (Nanohelix, Madison, WI, USA), according to the manufacturer’s instructions. BrightGreen qPCR master mix-ROX (ABM, New York, NY, USA) was used for PCR reactions. 

### 4.7. Western Blot

For western blot of phospho-ERK, minoxidil and PD98059 (Sigma-Aldrich) was treated for 30 min–1h. Cells were lysed with protein extraction solution (PRO-PREP^TM^; iNtRON, Seoul, Korea) containing phosphatase inhibitor (Na_3_VO_4_; Roche, Pleasanton, CA, USA). Western blot analysis was performed as described previously [8] using the following primary antibodies: rabbit anti-p42/44 (1:1500; Cell Signaling Technology, Danvers, MA, USA), mouse anti-phospho-p42/44 (1:1500; Cell Signaling Technology), and mouse anti-α-tubulin (1:2000; Santa Cruz Biotechnology, Dallas, TX, USA). Western blot images were obtained using ImageQuant LAS 4000 (GE Healthcare Life Science, Pittsburgh, PA, USA). 

### 4.8. Animal Experiment 

Mice were maintained and anesthetized according to a protocol that was approved by the US Pharmacopoeia and the Institutional Animal Care and Use Committee of Yonsei University (IACUC120002, 17 July 2015). The dorsal area of 6.5-week-old C3H/HeN mice in the telogen stage of the hair cycle was shaved with a clipper and electric shaver, with special care taken to avoid damaging the bare skin. Naïve ASCs or minoxidil-treated ASCs were only once injected into the dorsal skin of shaved mice. The growth factors (100 ng/mL per one day) were injected into the dorsal skin of shaved mice every day for 12 days. CXCL1 (R and D Systems, Minneapolis, MN, USA), PD-ECGF (R and D Systems) and PDGF-C (PeproTech, Rocky Hill, NJ, USA) proteins were purchased from each companies. For application with 2% minoxidil, CXCL1, PD-ECGF or PDGF-C (1 µg/mL) with 2% minoxidil (Rogaine; Johnson and Johnson Healthcare Products, Skillman, NJ, USA) were applied on the dorsal skin of shaved mice. Any darkening of the skin (indicative of hair cycle induction) was carefully monitored by photography. After 14 days, the dorsal hair was shaved and weighed to estimate growth rate [3,5].

### 4.9. Vibrissae Follicle Organ Culture

For organ culture of vibrissae hair follicle, the vibrissae hair follicle was cut from C3H/HeN mice, washed with PBS and cultured in defined medium (Williams E medium supplemented with 2 mM l-glutamine, 10 µg/mL insulin, 10 ng/mL hydrocortisone, 100 U/mL penicillin, and 100 µg/mL streptomycin, without serum) including CXCL1 (5 ng), PD-ECGF (20 ng) and PDGF-C (20 ng) for three days.

### 4.10. BrdU Labeling 

For BrdU labeling assay, 4 × 10^4^ cells were seeded in six well plates and incubated for 3 days after adding three proteins. BrdU (Sigma-Aldrich) was added in cell culture media to a final concentration of 200 mM and incubated for 4 h at 37 °C with 5% CO_2_. The cells were fixed with 4% paraformaldehyde, incubated with mouse anti-BrdU (1:500) (Abcam, Iowa City, IA, USA) overnight at 4 °C, and then incubated with secondary antibodies, Alexa Fluor 488 goat anti-mouse IgG (Invitrogen), for 1 h at room temperature with 4,6-diamidino-2-phenylindole (DAPI) (Sigma-Aldrich). Immunofluorescence staining was imaged using ZEISS LSM700 confocal microscope (Carl ZEISS, Oberkochen, Germany). For calculation of the percentage of BrdU^+^ cells, we counted the number of BrdU^+^ cells and DAPI^+^ cells (all cells) in every same size pictures using Adobe photoshop CS6 extended program (Yonsei University, Seoul, Korea), and calculated the percentage of BrdU^+^ cells.

### 4.11. Hematoxylin/Eosin and Immunofluorescence Staining

For Hematoxylin and eosin staining, paraffin sections were dewaxed using xylene for 30 min, hydrated in 100%-, 90%-, 80%- and 70% EtOH and was dipped into Mayer’s hematoxylin (Sigma-Aldrich) for 8 min, and then rinsed in water for 1 minute. Slide was dipped again into eosin Y (Sigma-Aldrich) for 80 s, dehydrated with 70%-, 80%-, 90%- and 100% EtOH, washed with fresh xylene for 30, dried, and mounted with mounting medium. Immunofluorescence staining was performed using standard protocols. Briefly, paraffin sections were dewaxed using xylene for 30 min, hydrated in 100%-, 90%-, 80%- and 70% EtOH and antigen retrieval was performed by boiling using microwave in antigen retrieval buffer (Dako, Carpinteria, CA, USA) for 2 min. The sections were treated with rabbit Ki67 antibody (1:300) (abcam) overnight at 4 °C, and were then incubated with secondary antibodies, Alexa Fluor 488 goat anti-rabbit IgG (Invitrogen), for 1 h at room temperature with 4,6-diamidino-2-phenylindole (DAPI) (Sigma-Aldrich). Immunofluorescence staining was imaged using ZEISS LSM700 confocal microscope. 

### 4.12. Statistical Analysis

All of the experiments were performed more than three times with independent cultures. Data are presented as mean ± standard error (SEM). Means were compared by Student’s *t*-test. For all statistical tests, a 0.05 level of confidence was accepted as statistically significant.

## Supplementary Materials

Supplementary materials can be found at http://www.mdpi.com/1422-0067/19/3/691/s1.

## Abbreviations

ASC	Adipose-derived stem cell
CXCL1	chemokine (C-X-C motif) ligand 1
PD-ECGF	platelet-derived endothelial cell growth factor
PDGF-C	platelet-derived growth factor-C
