# Supplementary material for: Minoxidil Promotes Hair Growth through Stimulation of Growth Factor Release from Adipose-Derived Stem Cells
Source: Int J Mol Sci. 2018 Feb 28;19(3):691. doi: 10.3390/ijms19030691 (PMC5877552; doi:10.3390/ijms19030691)
Supplement: Supplementary file 1 [file ijms-19-00691-s001.pdf]

International Journal of
Molecular Sciences

Supplementary Materials: Minoxidil Promotes Hair Growth through Stimulation of Growth Factor Release from Adipose-Derived Stem Cells

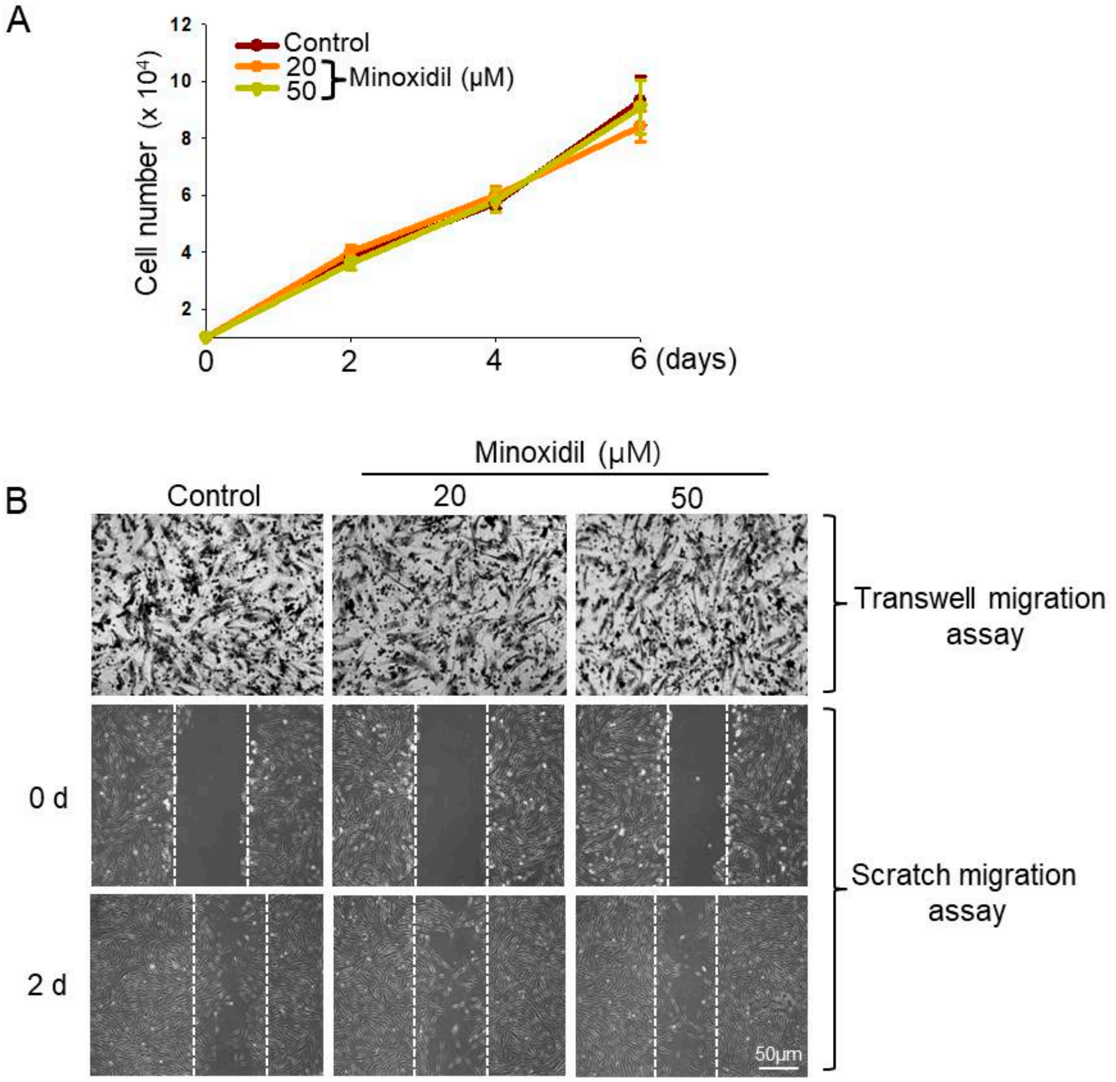

Figure S1. Minoxidil doesn't promote hDFs proliferation and migration. No effect of minoxidil on proliferation (**A**) and migration assay (**B**) of human dermal fibroblasts.

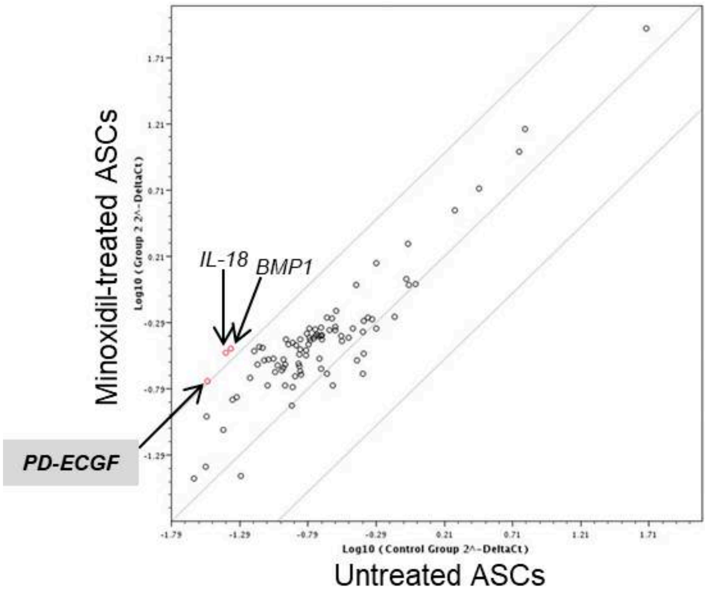

Figure S2. QPCR array for growth factors between minoxidil-untreated and -treated ASCs. Upregulation of three growth factors in minoxidil-treated ASCs was analyzed by QPCR array. Gray straight lines indicate ± 2-fold change. The three red circles indicate upregulated genes over 2-fold.

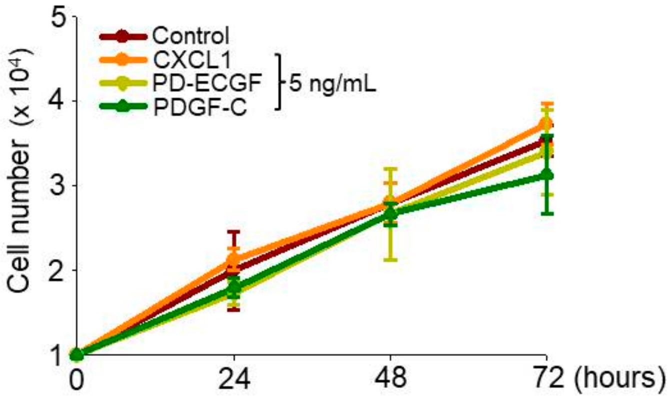

Figure S3. CXCL1, PD-ECGF, and PDGF-C doesn't induce proliferation of hDFs. No effect of three proteins (5ng) on proliferation of human dermal fibroblasts.
